# Maternal β-carotene addition has long-term effects on intestinal health of offspring chicks

**DOI:** 10.3389/fmicb.2025.1623816

**Published:** 2025-08-21

**Authors:** Taiping Wang, Da Wan, Tianyu Ren, Ye Tao, Min Wu, Xin Zheng

**Affiliations:** ^1^College of Animal Science and Technology, College of Veterinary Medicine, Jilin Agricultural University, Changchun, Jilin, China; ^2^Key Laboratory of Animal Production, Product Quality and Security, Ministry of Education, Jilin Agricultural University, Changchun, China

**Keywords:** β-carotene, maternal nutrition, gut health, offspring chicks, intestinal microbiota

## Abstract

**Background:**

Maternal dietary intervention utilizing complex additives rich in β-carotene has demonstrated the capacity to enhance embryonic intestinal development and influence microbial composition in offspring. Nevertheless, the extended impact of maternal β-carotene inclusion on the intestinal health of post-hatching chicks is still not fully elucidated.

**Objective:**

This research aimed to evaluate the impacts of maternal β-carotene supplementation on the intestinal development and microbial communities in chicks after hatching.

**Methods:**

A total of 1,215 Hy-Line Brown laying hens were divided into three groups, with each group containing five subgroups of 81 hens each. The hens received a basal diet (CON) or diets added with β-carotene at 120 mg/kg (LBC) or 240 mg/kg (HBC). After 6 weeks of dietary intervention, eggs were collected for incubation. Following hatching, 40 male chicks from each group were randomly chosen and evenly distributed into five distinct subgroups. All chicks were then reared on a uniform basal diet for a duration of 6 weeks.

**Results:**

Chicks from the LBC group exhibited significantly higher initial body weight, enhanced villus height (VH), greater villus height to crypt depth ratio (VCR), higher goblet cell (GC) density, and elevated expression of Mucin 2 (*MUC2*), zonula occludens-1 (*ZO1*), and zonula occludens-2 (*ZO2*) in both the jejunum and ileum at 42 days of age. In addition, maternal inclusion of β-carotene 240 mg/kg markedly improved VCR in the jejunum and ileum of chicks. In spite of the observed enhancements in intestinal health, there were no notable variations in overall growth performance across the groups during days 1 to 42. Beta diversity analysis revealed distinct microbial clustering in the ileum of both LBC and HBC groups, clearly separated from the CON group. Taxonomic profiling showed an enrichment of *Marivita, Burkholderia*, and *Turicibacter* in the CON group; *Oxalobacteraceae*, *Anoxybacillus*, *Roseburia*, and *Anaerorhabdus* in the LBC group; and *SMB53* and *Allobaculum* in the HBC group. Correlation analyses indicated positive associations between *Anaerorhabdus*, *Anoxybacillus*, and *Roseburia* and improved intestinal histomorphology, GC abundance, and barrier-related gene expression.

**Conclusion:**

These findings suggest that maternal β-carotene addition confers sustained benefits to intestinal health in offspring, potentially mediated by modulation of the cecal microbiome up to day 42.

## 1 Introduction

In animals, maternal factors influence the development and immune development of the offspring, thereby affecting progeny health and survival (Gomez De Agüero et al., 2016; Cheynel et al., 2021). In mammals, maternal nutrition and immunity support for offspring development and growth are mediated by the placenta and milk (Albrecht and Arck, 2020). Maternal nutrition status is one of the critical determinants of progeny health outcomes through the placenta and milk (Edwards et al., 2021). Additionally, the maternal status regarding a specific nutrient influences offspring responses to that nutrient and exerts lasting (Chen et al., 2022). In poultry, females contribute a single investment in offspring growth and health through the composition of the eggs (Hincke et al., 2019). We previously demonstrated that nutritional interventions in breeder hens influence offspring intestinal development and health through microbe-mediated maternal effects, initiated during embryonic development and independent of egg weight (Gong et al., 2023). Accumulating evidence suggests maternal nutritional interventions affect early-life intestinal development and gut microbiota composition in offspring (Jiang et al., 2022; Dai et al., 2024). Vedder et al. (2023) reported that maternal effect transmitted via egg weight in Japanese quail rapidly diminish after chick hatching. For the gut development and health of post-hatch chicks, however, whether there could be long-term effects of nutritional treatments in hens remains unclear.

The intestine, the largest surface-area organ in vertebrates, functions as the key site for nutrient absorption and significantly contributes to immune regulation (Mowat and Agace, 2014). Effective nutrient absorption through the gastrointestinal tract is fundamental to ensuring optimal growth and development in animals. The efficiency of digestion and nutrient uptake depends on the intestinal surface area, which is influenced by structural parameters (Weaver et al., 1991; Cervantes, 2013). Consequently, intestinal development relies on intestinal weight, length, and villus morphology. Furthermore, as a digestive organ, intestine functions as a critical interface connecting the external and internal environments, frequently encountering diverse harmful substances and pathogens. Therefore, it comes into frequent contact with a broad spectrum of harmful substances and pathogens. The intestines’ barrier function directly correlates with the organ’s health in the exchange of substances (Di Vincenzo et al., 2024). The gut barrier comprises multiple components, including adherent mucus gel layers and intercellular tight junctions. Mucin 2 (MUC2), the primary mucin within the adherent mucus gel layers, is produced by goblet cells (GCs) (Gundamaraju and Chong, 2021). Zonula occludens (ZO) and occludin (OCLN), which function respectively as intracellular plaque proteins and transmembrane proteins, are essential constituents of tight junctions (Furuse et al., 1993; Shigetomi et al., 2023). The gut microbiota impacts intestinal nutrient absorption and modulates host immunity, ultimately influencing overall health (Saint-Martin et al., 2024).

β-Carotene occurs mainly in dark fruits and vegetables. As a provitamin A additive in animal production, it has been found to enhance growth and development, improve immunity, regulate the gut flora, and promote the transfer of maternal immune factors via breast milk (Nishiyama et al., 2011; Wang et al., 2017; Li et al., 2021). Increasing evidence indicates that dietary provision of β-carotene in avian species contributes to health maintenance by improving immunity and antioxidant status. Hui et al. (2020) documented that β-carotene enhances intestinal development and immunity by increasing the population of beneficial bacteria. Moreover, it elevates immune factor levels in breeder eggs, thereby conferring health benefits to offspring (Cucco et al., 2006). However, it remains unclear whether adding β-carotene to maternal diets enhances intestinal development and modulates intestinal microbiota in chicks. We hypothesize that dietary inclusion of β-carotene in breeder hen enhances offspring health by promoting offspring intestinal development and modulating gut microbiota through maternal effects. Thus, the present research sought to quantify the role of maternal β-carotene supplementation in influencing the intestinal development and microbiota of chicks, thus assessing whether maternal β-carotene has long-term impacts on chick health post-hatching.

## 2 Materials and methods

### 2.1 Birds, treatments, and management

A total of 1,215 laying breeder hens (Hy-Line Brown, aged 45 weeks) were randomly assigned to three groups: control (CON) group, low β-carotene (LBC, 120 mg/kg) group, and high β-carotene (HBC, 240 mg/kg) group. Each group consisted of five subgroups containing 81 hens each. Basal diets were formulated according to GB/T 5916-2020 guidelines (Table 1). All hens were kept in cages, each comprising nine units with three hens per unit. Each unit was equipped with two nipple drinkers, and every set of three units on the same layer shared a feeder. Following a 7-day adaptation, the formal experiment began and lasted 6 weeks. Hens were provided *ad libitum* access to feed and water throughout the trial and were artificially inseminated every 5 days in the afternoon using semen collected from healthy Hy-Line^®^ Brown breeders. The controlled environmental conditions included 16 h of artificial light daily at 30 lux, temperatures between 23°C and 26°C, and humidity levels between 50% and 65%. Breeder eggs were collected from each group during the last 3 days of the hens feeding experiment and incubated following standard commercial hatchery conditions.

**TABLE 1:** Composition and nutrient levels of basal diets for laying breeder hens and chicks.

Items	Laying breeder hen diet	Chick diet
**Ingredient (%)**		
Corn	60.00	56.19
Soybean meal	22.30	19.61
Limestone	9.00	1.53
Wheat middling and red dog	3.00	8.00
Soybean oil	1.50	–
Puffed soybean	–	8.00
Corn gluten meal	–	2.00
Fish meal	–	2.00
CaHPO_4_	1.50	1.10
Premix^1^	1.40	1.00
Choline chloride	0.45	0.06
NaCl	–	0.25
Lys	0.35	0.15
Thr	0.30	0.10
Met	0.20	0.01
Total	100	100
**Calculated composition^2^ (%)**		
ME(KJ/kg)	11.22	12.90
CP	15.85	20.20
Ca	3.76	1.05
Available phosphorus	0.460	0.380
Lys	1.100	1.128
Methionine	0.430	0.487
Met + Cys	0.726	0.847

^1^The premix provided the following per kg of diet: vitamin A 8,500 IU, vitamin D 3,000 IU, vitamin E 30.0 IU, vitamin K_3_ 3.0 mg, vitamin B_2_ 12.5 mg, vitamin B_6_ 4.0 mg, vitamin B_12_ 20.0 mg, pantothenic acid 18.0 mg, niacin 60.0 mg, folic acid 1.5 mg, biotin 20.0 mg, Fe 80.0 mg, Cu 5.0 mg, I 0.6 mg, Se 0.3 mg, Mn 90.0 mg, and Zn 45.0 mg. ^2^Values were calculated based on data from the China Feed Database (2013).

At hatching, 40 male chicks from each group were selected and divided into five subgroups, with eight chicks per subgroup. All chicks were fed an identical basal diet for 6 weeks post-hatch. The dietary nutritional composition of the offspring diet is detailed Table 1. Chicks had *ad libitum* access to feed and water throughout the experiment. For the first 3 days, the brooding room was kept at 36°C; for days 4 and 7, it was dropped to 34°C; then from days 36 through 42, it was lowered by 2°C weekly until it stabilized at 24°C. Relative humidity was kept at approximately 60% from days 1 to 14, and then reduced to 50%. Lighting (LED) was provided continuously for 22 h per day during the first 3 days, subsequently reduced by 2 h each week from days 4 to 7, and finally stabilized at 12 h per day from days 36 to 42.

### 2.2 Growth performance

Chicks from each replicate group were weighed twice, first a hatching and again at 42 days of age. Data on feed consumption, final weight (FW), and initial weight (IW) were recorded. Based on these measurements, the average daily gain (ADG), the average daily feed intake (ADFI), and the feed-to-gain ratio (F/G) were computed. Since no chicks died during the experiment, mortality rate was not analyzed.

### 2.3 Sampling

At 42 days of age, 10 chicks were selected from each group for sampling, with two chicks per subgroup. The chicks were euthanized, and their intestines were subsequently isolated. The natural length and weight of the intestines were measured. For morphological and GC analyses, segments (approximately 3 cm) from the duodenum, jejunum, and ileum were gathered and then preserved in 4% paraformaldehyde. For gene expression analysis, the jejunum and ileum tissues were collected, washed with phosphate-buffered saline, rapidly frozen in liquid nitrogen, and subsequently kept at −80°C. Cecal contents were rapidly transferred to sterile cryogenic tubes and stored in liquid nitrogen until used for gut microbiota analysis.

### 2.4 Intestinal morphology

Following fixation in 4% paraformaldehyde, intestinal samples underwent trimming, dehydration, clearing, and paraffin embedding. Hematoxylin and eosin (H&E) were employed to stain the embedding tissues after they were cut into slices with a thickness of approximately 5 μm. Morphology was analyzed using a light microscope (Olympus BX51). Images were taken at 40× magnification with a camera (Olympus DP74, Tokyo, Japan), and Olympus Cell Stand imaging software (Tokyo, Japan) was used to make measurements. The villus height (VH) and crypt depth (CD) were measured from 15 intact villi per section following methods described by Wang et al. (2021), and the VH to CD ratio (VCR) was calculated.

### 2.5 PAS staining

Jejunal and ileal tissues were stained according to the Periodic Acid-Schiff (PAS) staining protocol. Samples were examined at 400× magnification and the GC number per 100 μm of villus was quantitated.

### 2.6 RNA extraction and qRT-PCR analysis

Total RNA of jejunum and ileum was isolated utilizing the RNAiso Plus Kit (9108Q, TaKaRa Biotechnology, Dalian, China). RNA purity and concentration were assessed with a NanoDrop-2000 spectrophotometer (Thermo Fisher Scientific, Waltham, MA, United States). RNA was converted to cDNA by reverse transcription using the PrimeScript RT Reagent Kit (RR047Q, TaKaRa Biotechnology, Dalian, China). The TB Green Premix Ex Taq II (RR420Q, TaKaRa Biotechnology, Dalian, China) was employed to real-time quantitative PCR (RT-qPCR) on a CFX Connect System (Bio-Rad, Hercules, CA, United States). Primer sequences are presented in Table 2. By utilizing GAPDH as an internal control, the expression of target genes may be normalized. Each sample was run three times to derive the levels of genes using the 2^ΔΔCt^ method.

**TABLE 2:** Primer sequences used for qPCR.

Target gene	Primer sequence (5′–3′)	Product size (bp)	Accession no.
*GAPDH*	F: CAGAACATCATCCCAGCGTCCAC	134	NM_204305.2
	R: CGGCAGGTCAGGTCAACAACAG		
*MUC-2*	F: TCACCCTGCATGGATACTTGCTCA	228	NM_001318434.1
	R: TGTCCATCTGCCTGAATCACAGGT		
*OCLN*	F: TCATCGCCTCCATCGTCTAC	240	NM_205128.1
	R: TCTTACTGCGCGTCTTCTGG		
*ZO-1*	F: TAAAGCCATTCCTGTAAGCC	243	XM_015278975.1
	R: GTTTCACCTTTCTCTTTGTCC		
*ZO-2*	F: GTTTCACCTTTCTCTTTGTCC	239	NM_204918.1
	R: TAAAGCCATTCCTGTAAGCC		

### 2.7 DNA extraction and 16S RNA sequencing

Total DNA of cecal contents was isolated utilizing the FastDNA^®^ SPIN Kit (MP Biomedicals, 11654600). The NanoDrop 2000 spectrophotometer and agarose gel electrophoresis were employed to ascertain the DNA’s concentration and purity. Utilizing barcoded fusion primers 338F (5′-ACTCCTACGGGAGGCAGCA-3′) and 806R (5′-GGACTACHVGGGTWTCTAAT-3′), the 16S rRNA gene’s V3–V4 section was amplified. The Agencourt AMPure XP Kit (Beckman Coulter, Indianapolis, IN, United States) was utilized for PCR amplicon purification. The Quant-iT PicoGreen dsDNA assay kit (Invitrogen, Carlsbad, CA, United States) was used for quantification. The purified amplicon was sequenced on an Illumina NovaSeq 6000 platform (Illumina, San Diego, CS, United States) with paired-end 2 × 250 bp reads.

### 2.8 Bioinformatics analyses

Bioinformatics analyses of sequencing data were performed using the QIIME2 pipeline (v2019.4) and R packages according to methodologies described previously (Gong et al., 2023). The primer sequence was systematically removed from the library utilizing the qiime cutadapt trim-paired command, and sequences that failed to align with designated primers were discarded. Subsequent quality filtering, denoising, sequence merging, and chimera removal were conducted using qiime dada2 denoise-paired. Following denoising, amplicon sequence variant (ASV) feature sequences without singletons were integrated into ASV tables. Singleton ASVs, defined as ASVs with a total count of 1 across all samples, were excluded from analysis. Taxonomic annotation of ASV sequences was conducted utilizing the SILVA database (v138) with a pre-trained Naive Bayes classifier in QIIME2, under default parameters (Quast et al., 2013; Bokulich et al., 2018). The ASV abundance table was subjected to rarefaction using the qiime feature-table rarefy function, with a depth set to 95% of the minimum sample size. Alpha diversity metrics at the ASV level were calculated using QIIME2. Employing principal coordinate analysis (PCoA), the beta diversity was assessed through the application of UniFrac distance metrics. Utilizing the taxonomy barplot plug-in, we conducted a count of the ASV feature table, omitting singletons, and employed bar charts to illustrate the taxonomic composition. By employing the UpSet package, we successfully generated visual representations that illustrate the shared and unique ASVs among different groups, while the VennDiagram package facilitated their identification. Differentially abundant taxa among groups were identified using Linear Discriminant Analysis Effect Size (LEfSe) with the default parameters (*P* < 0.05, LDA score > 2).

### 2.9 Statistical analysis

Data analyzed utilizing one-way ANOVA in IBM SPSS 26.0. Comparative analyses among groups were conducted utilizing Duncan’s test. The findings are presented as means ± standard error (SE). Spearman correlation analysis was used to evaluate the relationships between the microbial taxa and various gut-related parameters. Differences were considered significant when the *P* < 0.05. The outcomes were illustrated utilizing Prism 9 software. Significance relative to the CON group is indicated as follows: ns: *P* > 0.05, *P < 0.05, and **P < 0.01.

## 3 Results

### 3.1 Offspring chicks growth performance

As presented in Table 3, chicks hatched from hens supplemented with β-carotene exhibited significantly higher IW (*P* < 0.05). No notable changes were found among groups for FW at day 42 or for ADG, ADFI, or F/G ratios during days 1–42 day. Although intestinal length and weight tended to increase in the LBC and HBC groups, these differences were not statistically significant.

**TABLE 3:** Effects of maternal β-carotene inclusion on offspring chick growth performance (days 1–42) and intestinal parameters at day 42.

Item	CON	LBC	HBC
IW (g)^1^	38.19 ± 0.57^b^	40.79 ± 0.58^a^	39.23 ± 0.57^ab^
FW (g)^1^	634.96 ± 11.9	642.69 ± 18.97	650.91 ± 17.56
ADG (g)^1^	14.11 ± 0.38	14.69 ± 0.53	15.12 ± 0.39
ADFI (g)^1^	29.33 ± 0.52	27.93 ± 0.52	28.26 ± 0.49
F/G (g/g)^1^	2.09 ± 0.08	1.91 ± 0.08	1.87 ± 0.05
Intestinal weight (g)^2^	31.05 ± 2.08	35.87 ± 1.76	34.97 ± 0.83
Intestinal length (cm)^2^	118.9 ± 3.78	123 ± 4.18	123.46 ± 3.03

Different superscript letters within a row indicate significant differences (*P* < 0.05). IW, initial weight; FW, final weight; ADFI, average daily feed intake; ADG, average daily gain; F/G, feed-to-gain ratio. ^1^Each value indicates the average ± SE of five subgroups (*n* = 40). ^2^Each value indicates the average ± SE of 10 chicks.

### 3.2 Intestinal micromorphology in chicks

**FIGURE 1:**
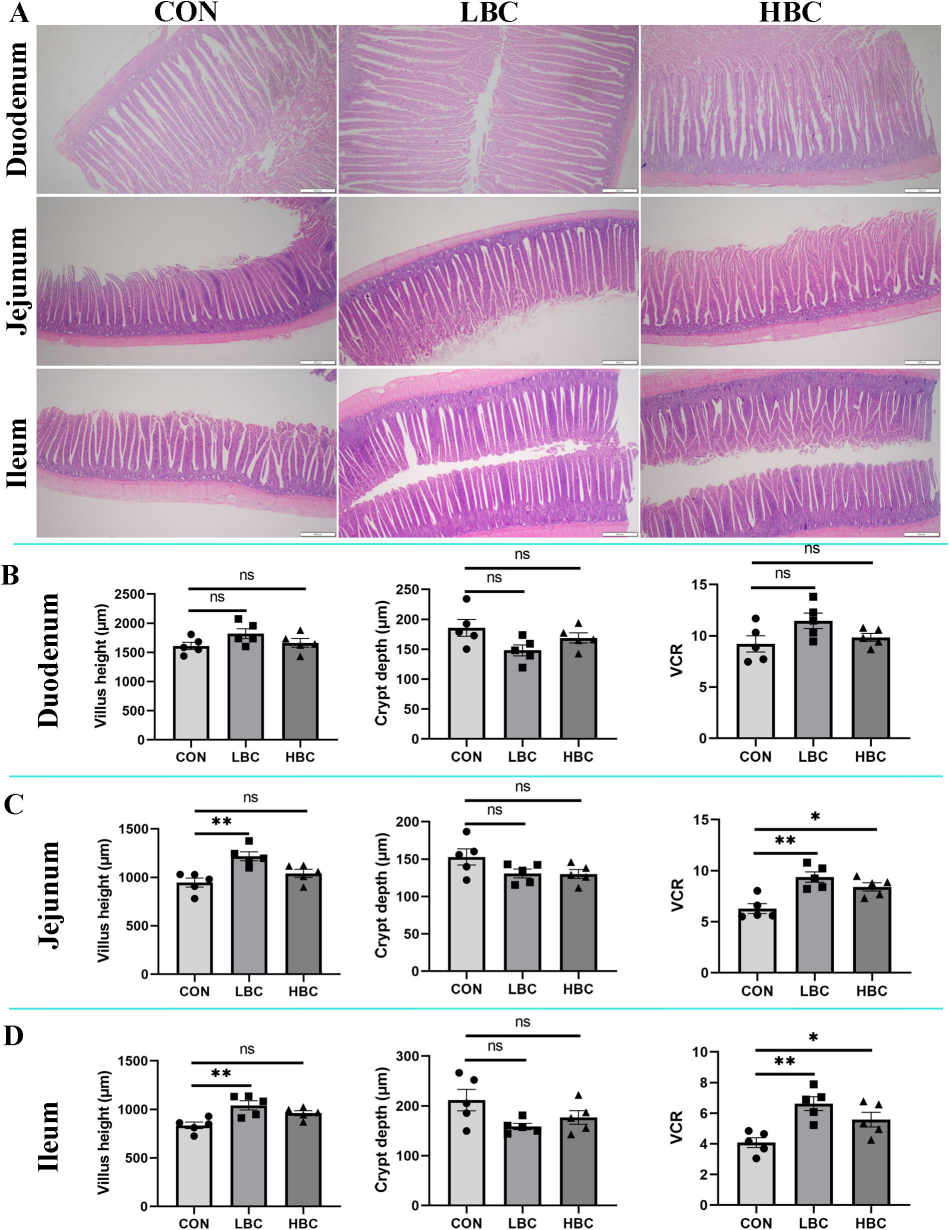
Effects of maternal β-carotene supplementation on small intestinal morphology in offspring chicks. **(A)** Histological sections of duodenum, jejunum, and ileum; **(B)** duodenum morphology; **(C)** jejunum morphology; **(D)** ileum morphology. CON, control group; LBC, hens consumed β-carotene at 120 mg/kg; HBC, hens consumed β-carotene at 240 mg/kg; VCR, villus height-to-crypt depth ratio. Data represent the mean ± SE of 10 chicks. Differences from CON group indicated as: *^ns^P* > 0.05, *P < 0.05, and **P < 0.01.

As illustrated in Figure 1, maternal β-carotene supplementation did not significantly alter VH, CD, or the VCR in the duodenum at day 42 (Figure 1B). However, chicks in the LBC group exhibited significantly greater (*P* < 0.01) VH and VCR in the jejunum and ileum, while no remarkable changes were noted in CD (Figures 1C, D). Additionally, the HBC group displayed significantly higher VCR values (*P* < 0.05) in both intestinal segments in comparison to the CON group.

### 3.3 GC density in the jejunum and ileum of chicks

**FIGURE 2:**
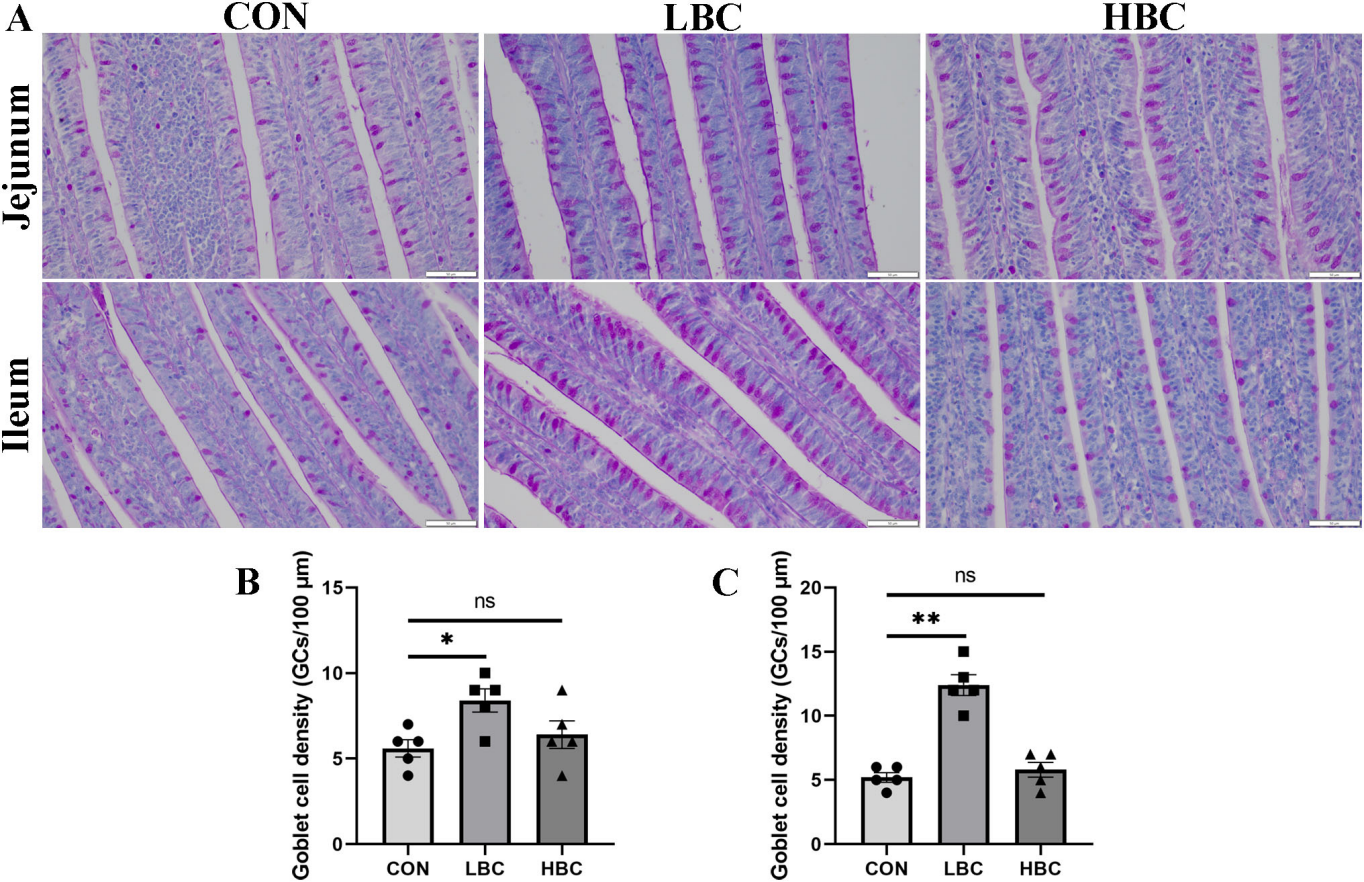
Effects of maternal β-carotene supplementation on GC density in offspring chick intestines. **(A)** PAS-stained images of GCs in jejunum and ileum; **(B)** jejunal GCs density; **(C)** ileal GCs density. CON, control group; LBC, hens consumed β-carotene at 120 mg/kg; HBC, hens consumed β-carotene at 240 mg/kg. Mean ± SE of 10 chicks. Significance vs. CON: *^ns^P* > 0.05, *P < 0.05, and **P < 0.01.

Through the application of PAS staining, we assessed the quantity of GCs present in the jejunum and ileum of chicks at 42 days (Figure 2). A marked increase (*P* < 0.05) in GC density was recorded in the LBC group, whereas no notable alterations were identified between the HBC and CON groups (Figures 2B, C).

### 3.4 Barrier gene expression in the jejunum and ileum of chicks

**FIGURE 3:**
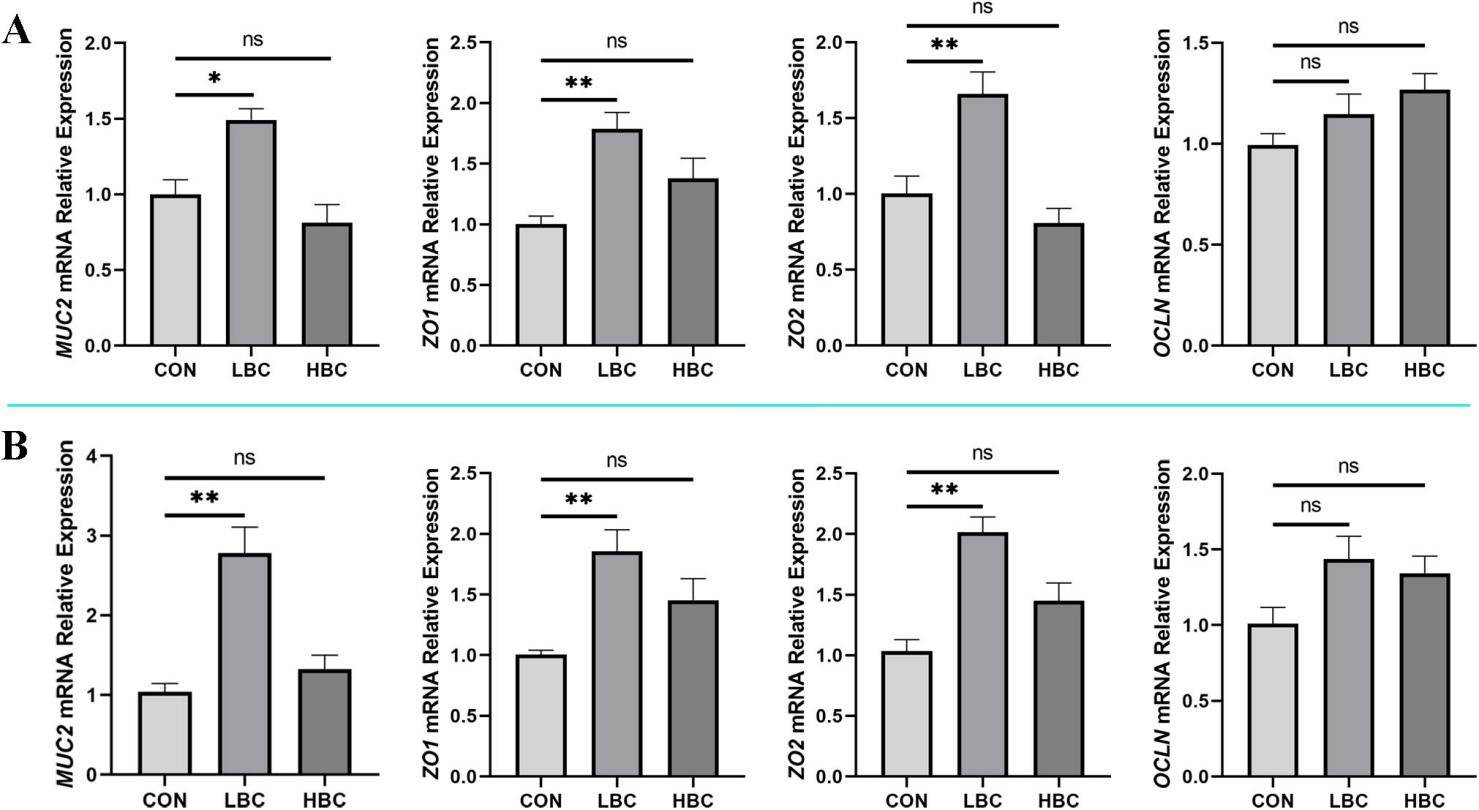
Effects of maternal β-carotene supplementation on intestinal barrier in offspring chicks. mRNA expression of *MUC2*, *ZO1*, *ZO2*, and *OCLN* in **(A)** jejunum and **(B)** ileum. CON, control group; LBC, hens consumed β-carotene at 120 mg/kg; HBC, hens consumed β-carotene at 240 mg/kg. *MUC2*, mucin-2; *ZO1*, zonula occludens-1; *ZO2*, zonula occludens-2; *OCLN*, occludin. Mean ± SE of 10 chicks. Significance vs. CON: *^ns^P* > 0.05, *P < 0.05, and **P < 0.01.

The influence of maternal β-carotene supplementation on epithelial barrier-related gene expression was evaluated at day 42 (Figure 3). Supplementation with 120 mg/kg β-carotene significantly enhanced (*P* < 0.05) the expression of *MUC2*, *ZO1*, and *ZO2*, while *OCLN* expression no significant change. In contrast, the administration of 240 mg/kg β-carotene did not notably influence the expression levels of any evaluated barrier-associated genes.

### 3.5 Microbial diversity analysis

**FIGURE 4:**
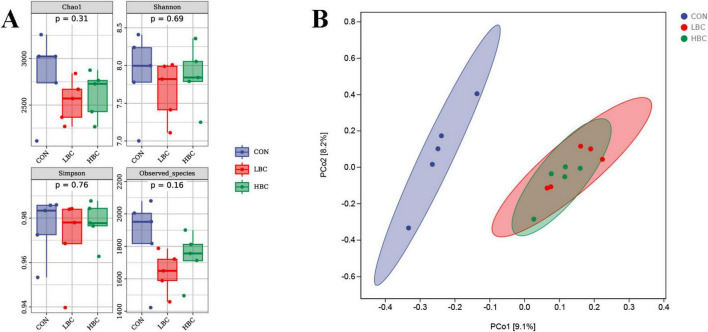
Effects of maternal β-carotene supplementation on cecal microbiota diversity in offspring chicks. **(A)** Alpha diversity indices. **(B)** Beta diversity visualized by principal coordinate analysis (PCoA).

As illustrated in Figure 4A, we assessed cecal microbiota alpha diversity in offspring chicks and observed no notable changes in the Chao1, Shannon, Simpson, or Observed_species indices when comparing the LBC and CON groups, as well as the HBC and CON groups.

The results of beta diversity analysis indicated that samples from the LBC and HBC groups clustered closely together, with partially overlapping 95% confidence intervals (CIs), and were clearly separated from the CON group (Figure 4B).

### 3.6 Taxonomic composition analysis

**FIGURE 5:**
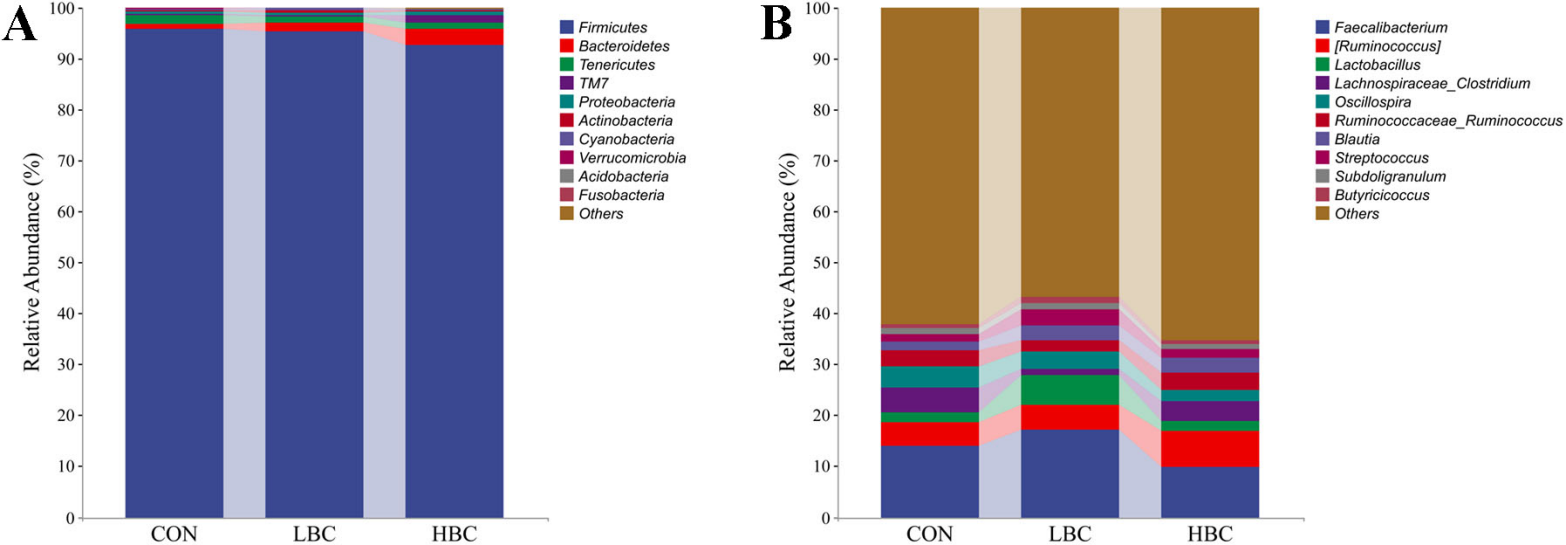
Effects of maternal β-carotene supplementation on the taxonomic composition of cecal microbiota in offspring chicks. Microbiota composition at the phylum **(A)** and genus **(B)** levels.

As illustrated in Figure 5A, the cecal microbiota of offspring chicks predominantly comprised three bacterial phyla: *Firmicutes*, *Bacteroidetes*, and *Tenericutes*. These phyla represented 98.47%, 98.32%, and 97.00% of the total bacterial population in the CON, LBC, and HBC groups, respectively. Firmicutes exhibited the highest relative abundance, accounting for 95.77%, 95.33%, and 92.69% in the respective groups.

Dominant genera included *Faecalibacterium*, *[Ruminococcus]*, *Lactobacillus*, *Lachnospiraceae_Clostridium*, *Oscillospira*, *Ruminococcaceae_Ruminococcus*, *Blautia*, and *Streptococcus*, collectively constituting 35.88%, 40.86%, and 32.98% of the total bacterial populations in the CON, LBC, and HBC groups, respectively (Figure 5B). The abundances of *[Ruminococcus]*, *Lactobacillus*, *Blautia*, and *Streptococcus* were elevated in the LBC and HBC groups, while *Lachnospiraceae_Clostridium* and *Oscillospira* exhibited decreased abundance. Additionally, *Faecalibacterium* abundance elevated in the LBC group but reduced in the HBC group, whereas *Ruminococcaceae_Ruminococcus* exhibited an inverse pattern, increasing in the HBC group but decreasing in the LBC group.

### 3.7 Analysis of microbiota differences among groups

**FIGURE 6:**
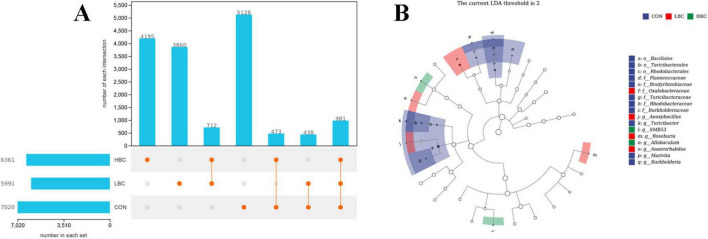
**(A)** Unique and shared taxa among groups. **(B)** Significantly enriched taxa from phylum to genus level, identified by LEfSe analysis [log_10_(LDA score) > 2, *P* < 0.05].

The total numbers of identified taxa in the CON, LBC, and HBC groups were 7,020, 5,991, and 6,361, respectively, with 5,128, 3,860, and 4,195 unique taxa per group (Figure 6A). A total of 438 taxa were shared between the CON and LBC groups, 473 taxa between the CON and HBC groups, and 981 taxa were common among all three groups. LEfSe analysis identified significantly enriched taxa among groups at the phylum-to-genus levels (Figure 6B). Specifically, 11 taxa, such as *Marivita*, *Burkholderia*, and *Turicibacter*, were markedly enriched (*P* < 0.05) in the CON group. In the LBC group, *Oxalobacteraceae*, *Anoxybacillus*, *Roseburia*, and *Anaerorhabdus* presented marked enrichment (*P* < 0.05). Furthermore, in the HBC group, *SMB53* and *Allobaculum* demonstrated notable enrichment (*P* < 0.05).

### 3.8 Correlation of cecal microbiota with intestinal development and barrier function

**FIGURE 7:**
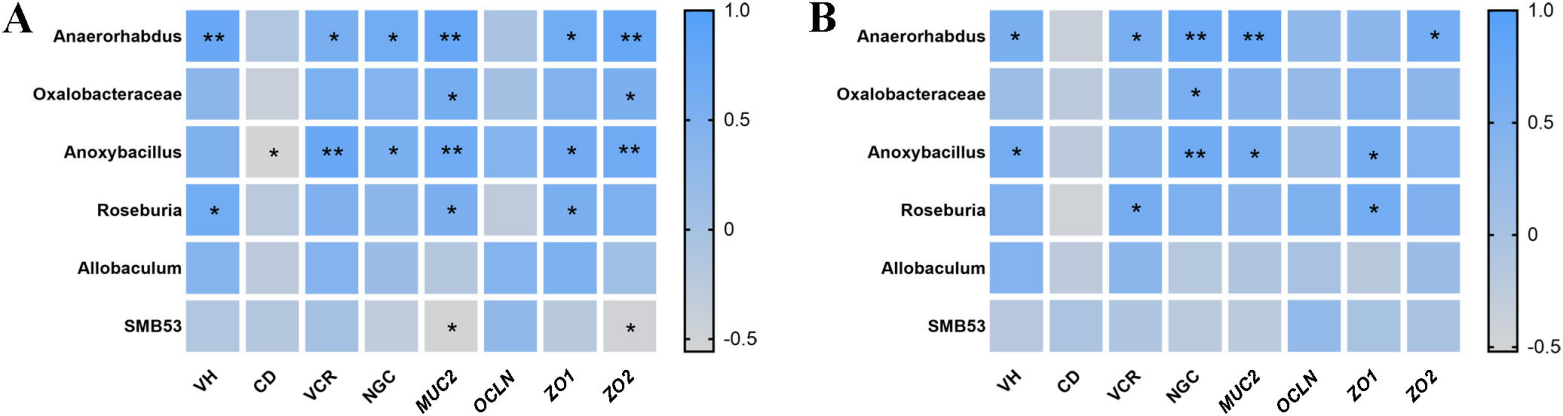
Spearman correlations between enriched cecal microbiota taxa and intestinal morphology/barrier function in offspring chicks (**A**, jejunum; **B**, ileum). VH, villus height; CD, crypt depth; VCR, the ratio of villus height to crypt depth; NGC, the number of goblet cells; *MUC2*, mucin-2; *ZO1*, zonula occludens-1; *ZO2*, zonula occludens-2; *OCLN*, occludin. *P < 0.05 and **P < 0.01.

To explore potential associations between cecal microbial taxa and intestinal morphological characteristics or barrier-related gene expression, Spearman correlation analysis was conducted (Figure 7). In the jejunum, *Anaerorhabdus* exhibited strong positive correlations with VH, VCR, and NGC, and the expression of *MUC2*, *ZO1*, and *ZO2*. *Anoxybacillus* displayed positive correlations with VCR, NGC, and the expression of *MUC2*, *ZO1*, and *ZO2*. *Roseburia* showed positive correlations with VH, as well as the expression of *MUC2* and *ZO1*. Additionally, *Oxalobacteraceae* exhibited positive correlations with *MUC2* and *ZO2* expression. *SMB53* exhibited negative correlations with *MUC2* and *ZO2* expression, while *Anoxybacillus* negatively correlated with CD. In the ileum, *Anaerorhabdus* remained positively correlated with VH, VCR, and NGC, and the expression of *MUC2* and *ZO2*. *Anoxybacillus* showed positive correlations with VH, NGC, and *MUC2* and *ZO1* expression. *Oxalobacteraceae* correlated positively with NGC, and *Roseburia* was correlated positively with VCR, and *ZO1* expression.

## 4 Discussion

Maternal influences contribute to offspring phenotype through mechanisms unrelated to genetic inheritance, referred to as maternal effects (Moore et al., 2019). Such effects can reflect maternal adaptations to the environment and modulate the developmental pathways of offspring (Groothuis et al., 2019). Maternal dietary supplementation with compounds such as stevioside, flavonoids from mulberry leaves, and multi-component additives like allicin and sodium butyrate has been observed to enhance offspring growth, immunity, intestinal development, and gut microbiota composition (Gong et al., 2020a; Jiang et al., 2022; Gong et al., 2023; Huang et al., 2024). Dietary supplementation with β-carotene in breeding birds has been shown to increase egg lysozyme concentrations and promote progeny growth and liver development (Cucco et al., 2006; Wang et al., 2023; Wang et al., 2024). Our findings indicate that diets supplemented with β-carotene in breeder hens significantly improved the IW of hatched chicks. This observation aligns with results from Gong et al. (2020b), who documented that hen diets enriched with additive blends notably enhanced offspring IWs. These findings indicate that consumption of β-carotene in hen diets exerts positive effects on progeny chick growth and development. However, the intervention did not significantly affect chick growth performance from days 1 to 42, nor did it influence the FW or the intestinal weight and length at day 42, although upward trends were noted. In alignment with our findings, Wang Y. et al. (2020) documented that maternal dietary inclusion of vitamin A improved offspring growth performance up to 21 days of age but not thereafter. The findings suggest that the beneficial impact of maternal β-carotene supplementation on offspring growth performance does not persist to 42 days of age. A previous study demonstrated that maternal effects induced by maternal diet in Japanese quail diminish rapidly as offspring age (Vedder et al., 2023). Accordingly, we speculate that the disappearance of the early weight advantage in offspring chicks may be attributed to the declining maternal effect. Moreover, further investigation is needed to determine whether compensatory growth in offspring contributes to the loss of this early weight advantage.

The gut serves as the primary site for nutrient digestion and absorption, significantly influencing poultry growth performance through its structural characteristics and barrier function (Cheled-Shoval et al., 2011). Previous research has supported that β-carotene supplementation effectively improves intestinal architecture and barrier function in layer-type cockerels at various developmental stages (7, 14, 21, and 28 days), while also enhancing growth performance during early life (Hui et al., 2020). In this research, maternal β-carotene supplementation improved the VH and VCR of jejunum and ileum in progeny chicks at day 42; however, no significant morphological changes were noted in the duodenum. Prior studies suggest that intestinal morphological parameters, namely VH, CD, and VCR, are intricately associated with the efficiency of nutrient absorption and, as a result, the growth of the organism (Pluske et al., 1996; Geyra et al., 2001). Although morphological enhancements were observed, no remarkable variations were found in overall growth performance among the treatments by day 42. Pancreatic juice and bile, essential for digestion in the small intestine, are secreted into the duodenum, with pancreatic juice secretion stimulated by chyme entering this region. Therefore, we hypothesize that the absence of improvement in duodenal morphology might explain the lack of significant enhancement in growth performance despite morphological improvements in other intestinal regions. Nevertheless, Wang J. G. et al. (2020) demonstrated that injecting N-acetyl-L-glutamate into breeding eggs significantly improved intestinal morphology in chicks without affecting growth performance. Thus, the reason maternal β-carotene enhanced jejunal and ileal morphology without influencing chick growth performance from days 1 to 42 remains unclear and requires further investigation.

Subsequently, we assessed GC density and the gene expression related to barrier function in the jejunum and ileum. Results revealed that both GC abundance and mRNA expression of *MUC2*, *ZO1*, and *ZO2* were markedly elevated in the LBC group, indicating enhanced mucosal barrier integrity in offspring due to maternal β-carotene supplementation. GCs, highly polarized columnar epithelial cells, secrete a mucin layer that protects the intestinal mucosa and plays vital roles in microbial homeostasis and pathogen defense (Duangnumsawang et al., 2021). GCs first appear in the intestinal villi epithelium on embryonic day 18, undergoing rapid proliferation between days 18 and 21 (Al Amin et al., 2025). Alterations during developmental stages can have long-lasting effects on intestinal function (Van Zijderveld et al., 1992). Various factors regulate villus GC counts. For example, chicks fed a diet inclusion of 1% stevia exhibited significantly increased villus GC density (Medeot et al., 2023). Similarly, increased GC numbers were observed in offspring from breeder hens consuming nano-selenium at 0.30 mg/kg (Chen et al., 2024). MUC2, as the predominant element of the mucin layer, is essential for maintaining intestinal barrier integrity. MUC2 expression in GCs is a critical focus in regulating barrier function (Birchenough et al., 2016; Liu et al., 2023). ZO also vital for gut barrier restoration, as they modulate tissue fluidity and influence epithelial cell replication (Kuo et al., 2021; Skamrahl et al., 2021). In our experiments, *MUC2*, *ZO1*, and *ZO2* expression in the jejunum and ileum corresponded with observed GCs density. These results indicate that maternal dietary supplementation with β-carotene positively affects the intestinal health of chicks at 42 days of age by increasing GC density in jejunal and ileal villi and enhancing *MUC2*, *ZO1*, and *ZO2* expression. However, it should be noted that maternal supplementation with 240 mg/kg β-carotene did not significantly affect the GCs density or the expression of barrier-related genes in the jejunum and ileum of offspring. This suggests that there may be a threshold for the effect of maternal β-carotene supplementation on intestinal health in offspring. Therefore, when aiming to improve offspring health through maternal β-carotene supplementation, the dosage should be carefully selected.

Growing evidence indicates that intestinal health is influenced by gut microbiota, which affects GCs and barrier integrity (Deplancke and Gaskins, 2001). Our previous research showed that dietary supplementation of breeder hens with a complex additive improved gut development in offspring embryos, presumably through enhanced vertical transfer of maternal microbiota (Gong et al., 2023). However, little is known about the long-term impacts of maternal β-carotene consumption on the offspring’s gut microbiota. In our research, beta diversity analysis of microbiota at day 42, demonstrated that samples from the LBC and HBC groups clustered closely and were markedly detached from those of the CON group, implying a sustained impact of maternal β-carotene on the cecal microbiota of post-hatch chicks. At the level of genera, taxa enriched in the CON group were primarily Gram-negative bacteria like *Marivita* and *Burkholderia*, whereas taxa enriched in the LBC and HBC groups were predominantly Gram-positive. Specifically, the LBC group showed enrichment of *Anoxybacillus*, *Roseburia*, and *Anaerorhabdus*, whereas *SMB53* and *Allobaculum* were enriched in the HBC group. Previous research indicated that *Burkholderia* can cause various diseases in poultry and animals under certain conditions and potentially pose threats to human health (Höger et al., 2016). *Roseburia* produces short-chain fatty acids (SCFAs), which contribute positively to gut health (Han et al., 2024; Jiang et al., 2025). *Anaerorhabdus* and *Allobaculum* have the potential to act as probiotics with a positive correlation to SCFA (Xie et al., 2022; Lin et al., 2023), while *SMB53* has been correlated with potentially harmful microorganisms (Ma et al., 2023). These outcomes indicate that maternal β-carotene supplementation may improve offspring intestinal health by enriching beneficial microorganisms within the ileal microbiota and reducing potentially harmful zoonotic pathogens. Correlation analysis showed that *Anaerorhabdus, Roseburia*, and *Anoxybacillus* exhibited positive correlations with intestinal development, GC density, and barrier function, while *SMB53* negatively correlated with intestinal barrier function. Consistent with our findings, Guo et al. (2023) observed *Roseburia* abundance was positively correlated with the feed conversion ratio in broilers, where feed conversion ratio is closely associated with improved gut morphology. This concurs with the positive correlation between *Roseburia* and intestinal structure observed in the current study. Conversely, increased abundance of *SMB53* has been linked to higher oxidative stress (Li et al., 2019), intestinal morphological disruptions, and impaired barrier function (Wang et al., 2022; Tang et al., 2023). These findings may explain the negative correlation between *SMB53* and barrier function and the comparatively weaker improvement in GC quantity and barrier function observed in the HBC group. Overall, the present research confirms that maternal β-carotene supplementation modulates cecal microbiota composition, increases intestinal development, enhances GC density and barrier function, and ultimately enhances offspring intestinal health at 42 days of age. In addition, maternal supplementation with β-carotene improved intestinal structure and barrier-related gene expression, but it did not enhance chick growth performance. Whether maternal β-carotene enhances the stress-induced recovery capacity of offspring chicks through maternal effects remains to be further investigated.

However, this study has several limitations, primarily due to the absence of data on fertilization rates, hatching rates, and embryo mortality rates, which limited our ability to evaluate the impact of dietary β-carotene supplementation on the reproductive performance of laying hens. Furthermore, the gut microbiota of chicks undergoes significant developmental changes during early life, and static endpoint analysis is insufficient to capture the dynamic progression of microbial colonization during this critical period. Therefore, we recommend incorporating data on fertilization rates, hatching rates, and embryo mortality rates, as well as implementing multi-point longitudinal sampling for gut microbiota analysis in future studies, to more accurately characterize both the effects of maternal nutrition on offspring during early life and the temporal development of the gut microbiota in chicks.

## 5 Conclusion

In summary, maternal β-carotene supplementation produces long-term beneficial effects on gut health in offspring chicks through day 42 by increasing jejunal and ileal morphology, increasing GC density, strengthening barrier function, and modifying the cecal microbiota. More particularly, maternal β-carotene inclusion decreases the relative abundance of the potentially zoonotic avian pathogen *Burkholderia* within the cecal microbiota. Nevertheless, the specific mechanisms driving these effects are still not fully understood and necessitate additional research.

## Data Availability

The datasets presented in this study can be found in online repositories. The names of the repository/repositories and accession number(s) can be found below: https://www.ncbi.nlm.nih.gov/bioproject/PRJNA1257966.
